# Supplementary material for: Large-scale pan-cancer analysis reveals broad prognostic association between TGF-β ligands, not Hedgehog, and GLI1/2 expression in tumors
Source: Sci Rep. 2020 Sep 2;10:14491. doi: 10.1038/s41598-020-71559-w (PMC7468122; doi:10.1038/s41598-020-71559-w)
Supplement: Supplementary file 2 — Supplementary Figures. [file 41598_2020_71559_MOESM2_ESM.pdf]

Supplementary information

Large-scale pan-cancer analysis reveals broad prognostic association between TGF-β ligands, not Hedgehog, and GLI1/2 expression in tumors

Aurélien de Reyniès[1]*, Delphine Javelaud[2,3], Nabila Elarouci[1], Véronique Marsaud[2,3], Cristèle Gilbert[2,3], and Alain Mauviel[2,3]*

[1]Programme Cartes d'Identité des Tumeurs, Ligue Nationale Contre le Cancer, 14 rue Corvisart, 75013 Paris, France

[2]Institut Curie, PSL Research University, INSERM U1021, CNRS UMR3347, Team "TGF-ß and Oncogenesis", Equipe Labellisée LIGUE 2016, F-91400, Orsay, France

[3]Université Paris-Sud, F-91400, Orsay, France

*Co-corresponding authors: Dr Aurélien de Reyniès, Programme Cartes d'Identité des Tumeurs, Ligue Nationale Contre le Cancer, 14 rue Corvisart, 75013 Paris, France; P: +33 1 53 55 25 12; E-mail: Aurelien.DeReynies@ligue-cancer.net; and Dr Alain Mauviel, Team "TGF-ß and Oncogenesis", Institut Curie, University center, Bldg 110, 91405 Orsay cedex, France; P: +33 1 69 86 30 43; Fax : +33 1 69 86 30 51 ; E-mail: alain.mauviel@curie.fr

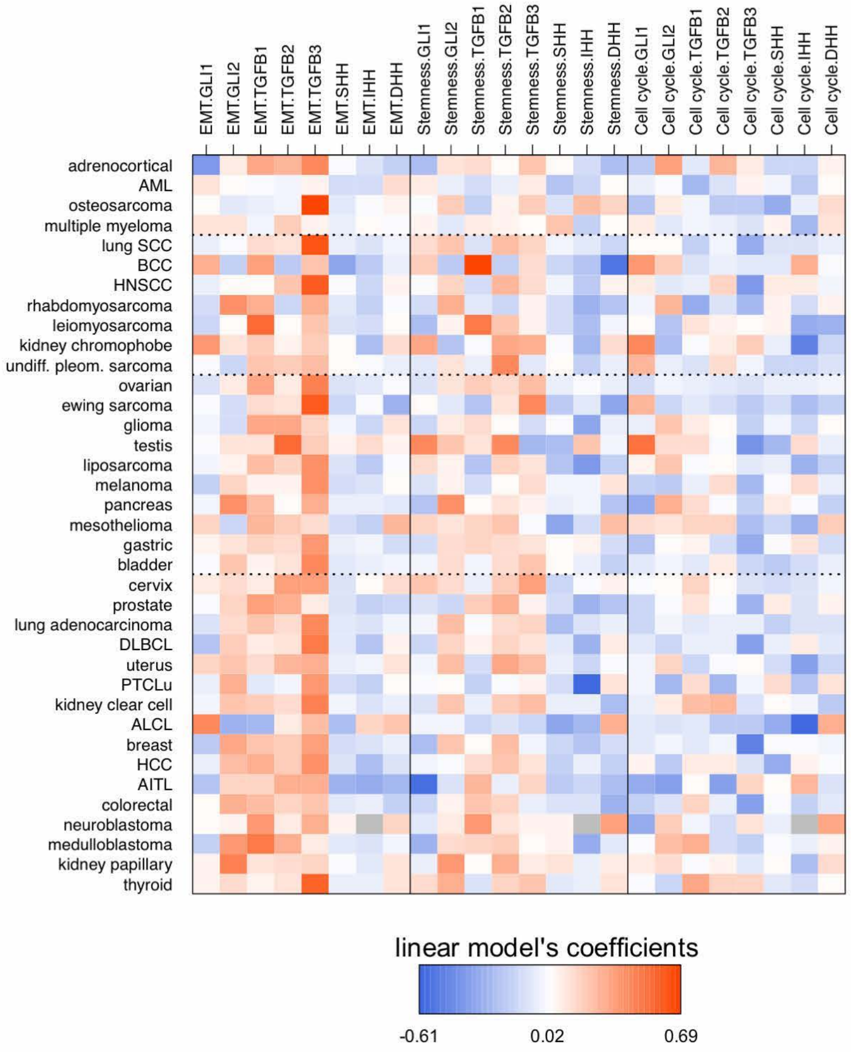

Supplementary Figure S1. Heatmap representation of the coefficients from the linear models generated for each of the 3 metagenes in each tumor type, based on the expression of *GLI1*, *GLI2*, *TGFB1*, *TGFB2*, *TGFB3*, *SHH*, *IDH*, *DHH*. The corresponding numerical values are provided in Supplementary Table S4B.

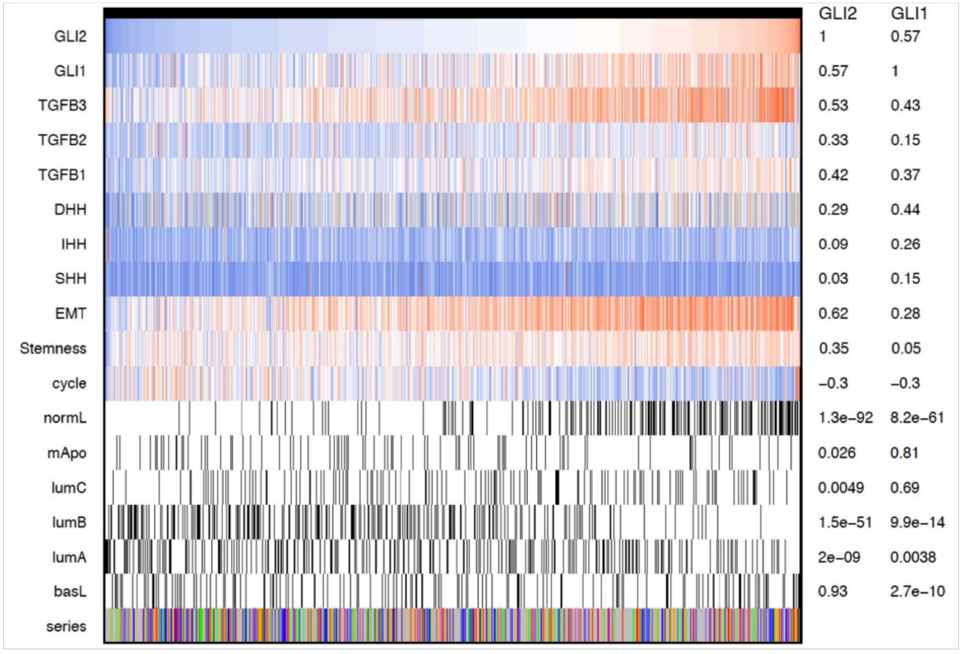

Supplementary Figure S2. Alignment of *GLI1*, *GLI2*, *SHH*, *DHH*, *IHH*, *TGFB1*, *TGFB2* and *TGFB3* as well as mesenchymal/EMT, Stemness and Cell cycle metagene expression profiles with molecular subtypes of breast carcinomas (6 series, 2556 patients).
